# Towards an anti-disease malaria vaccine

**DOI:** 10.1042/ETLS20170091

**Published:** 2017-12-22

**Authors:** Frank Lennartz, Thomas Lavstsen, Matthew K. Higgins

**Affiliations:** 1 Department of Biochemistry, University of Oxford, South Parks Road, Oxford OX1 3QU, U.K.; 2 Centre for Medical Parasitology, Department of Immunology and Microbiology (ISIM), Faculty of Health and Medical Sciences, University of Copenhagen, Copenhagen, Denmark; 3 Department of Infectious Diseases, Copenhagen University Hospital (Rigshospitalet), Copenhagen, Denmark

**Keywords:** malaria, surface protein, vaccine

## Abstract

Human infective parasites, such as those that cause malaria, are highly adapted to evade clearance by the immune system. In situations where they must maintain prolonged interactions with molecules of their host, they often use parasite surface protein families. These families are highly diverse to prevent immune recognition, and yet, to promote parasite survival, their members must retain the ability to interact with specific human receptors. One of the best understood of the parasite surface protein families is the PfEMP1 proteins of *Plasmodium falciparum*. These molecules cause infected erythrocytes to adhere to human receptors found on blood vessel and tissue surfaces. This protects the parasite within from clearance by the spleen and also causes symptoms of severe malaria. The PfEMP1 are exposed to the immune system during infection and are therefore excellent vaccine candidates for use in an approach to prevent severe disease. A key question, however, is whether their extensive diversity precludes them from forming components of the malaria vaccines of the future?

## Do anti-disease immunogens have a place in future malaria vaccines?

Malaria is one of the most ancient diseases of humanity, evolving with its mammalian hosts for millennia. It still kills around half a million people each year and causes hundreds of millions of clinical cases [1]. The quest to generate a vaccine has been long and challenging, with the best example to date, the single-component vaccine RTS,S, proving to be only ∼30% effective [2]. The malaria vaccines of the future are therefore likely to become more complex, simultaneously attacking multiple stages of the parasite life cycle [3,4]. The three major targets currently leading the way are the molecular machinery used by the parasite to invade human liver cells, to invade red blood cells or to fuse gametes in the mosquito midgut. Effectively preventing any one of these crucial steps in the parasite life cycle will block parasite development or will stop transmission to other human hosts [5]. One option, which leads to sterile protection in immunized volunteers, but has challenges associated with deployment in malaria endemic regions, is whole parasite-based vaccines, such as attenuated sporozoites [6]. In addition, promising molecular targets for intervention have been identified for each stage, including components of the circumsporozoite protein (CSP) for the liver stage (already the active component of the RTS,S vaccine) [2], RH5 for the blood stage [7,8] and HAP2, Pfs48/45 and Pfs230 for gamete fusion [9]. All are under active consideration as vaccine candidates.

Other possibilities on the target list are the proteins that determine whether a malaria infection develops severe and life-threatening symptoms. Severe malaria, which may include severe anaemia, respiratory distress or cerebral effects, is experienced among individuals who have not yet been exposed to repeated *Plasmodium falciparum* infections and have therefore not developed an antibody repertoire capable of managing the infection [5]. In malaria endemic areas, the burden of severe malaria falls heaviest on children under the age of 5 [10], while as transmission decreases, immunity is acquired later in life, and severe malaria becomes more common in older children [5,11].

**Figure 1.**
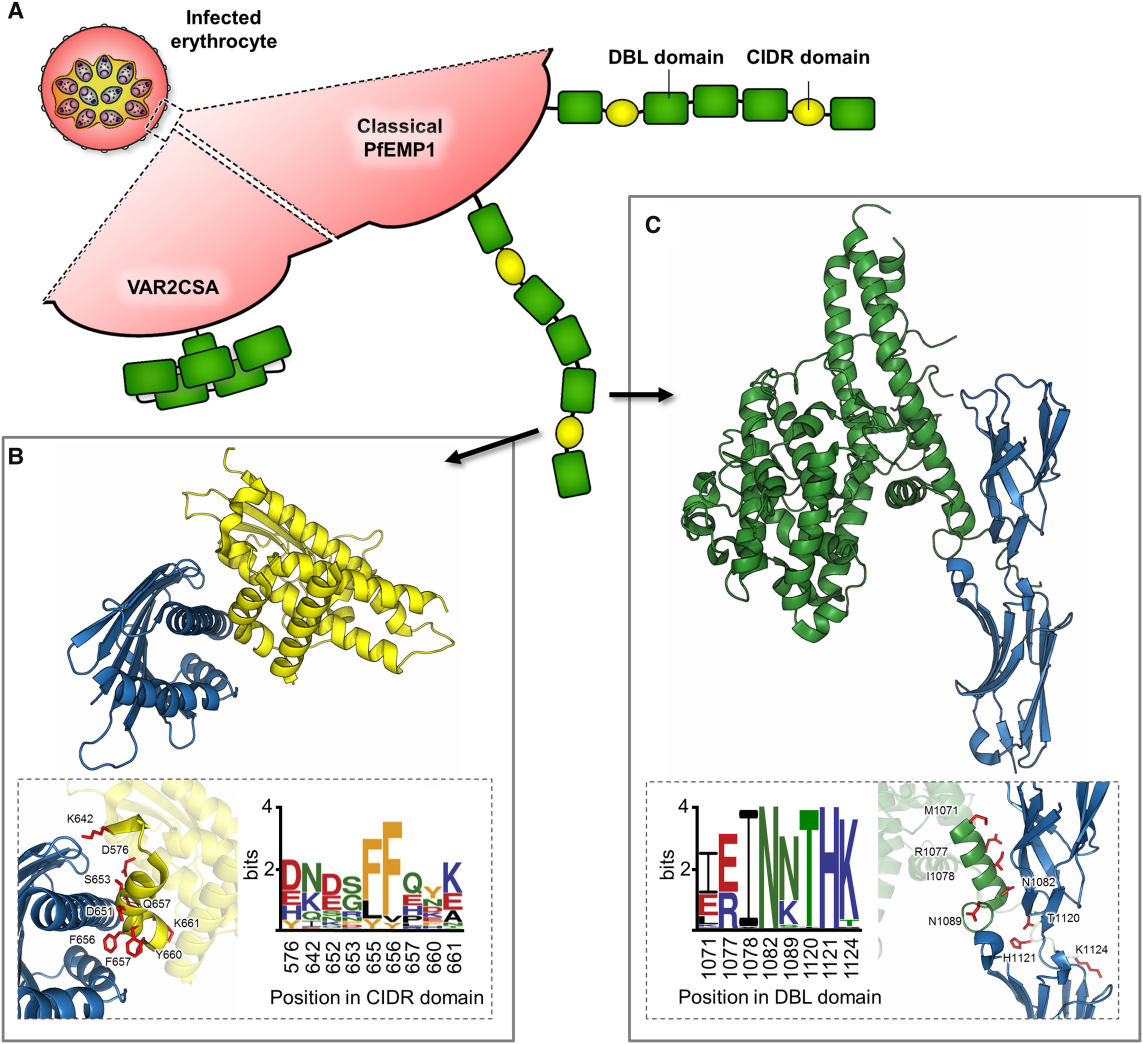
The molecular basis for binding of PfEMP1 associated with severe disease to human receptors. (**A**) The architecture of PfEMP1 ectodomains displayed on the surface of erythrocytes infected with *P. falciparum*. Classical PfEMP1s are rigid and elongated in structure [51], whereas VAR2CSA is the only PfEMP1 known to have an overall globular structure [52]. The two domain types in PfEMP1, CIDR domains (yellow) and DBL domains (green), are indicated. (**B**) Crystal structure of a CIDR domain (yellow) bound to its human receptor, EPCR (blue). The inset shows the CIDR residues (red) that make direct contact with EPCR and their conservation across 737 EPCR-binding CIDR domains, displayed as a sequence logo [36]. (**C**) Crystal structure of a DBL domain (green) bound to the first two domains of its human receptor, ICAM-1 (blue). The inset shows the DBL residues (red) that directly contact ICAM-1 and a sequence logo showing their conservation across 145 ICAM-1-binding DBL domains [38].

The development of severe malaria symptoms occurs in the blood stage of infection and is linked to expression of parasite proteins found on infected erythrocyte surfaces, tethering parasites to human endothelium and tissues. This prevents infected erythrocytes, which have been swollen by parasites within, from being filtered from the blood by the human spleen. It also leads to inflammation, occlusion of blood vessels and to the symptoms of severe malaria, including cerebral malaria and pregnancy-associated malaria, which are characterized by marked sequestration of parasites in the brain and placenta [12]. Endothelial tethering is mediated by the *P. falciparum* erythrocyte membrane protein 1, PfEMP1, a large protein family with ∼60 members encoded in each genome [13,14]. Each PfEMP1 is formed from multiple copies of two parasite-specific domain types, the CIDR and DBL domains, which are most often spatially arranged in a linear array (Figure 1) [15]. Different individual domains are capable of interacting with specific human endothelial surface proteins, including endothelial protein C receptor, EPCR [16], intracellular adhesion molecule 1, ICAM-1 [17] and cluster of differentiation 36, CD36 [18].

In some regards, the PfEMP1s are excellent vaccine candidates. They are linked directly to the pathogenesis of malaria, are constantly exposed to the immune system and are the targets of many of the antibodies generated in response to infection [19,20], and that correlate with protection against malaria [21]. A vaccine that raises antibodies that target PfEMP1s and prevents endothelial adhesion is therefore expected to significantly reduce the incidence of severe symptoms and deaths due to malaria.

However, the PfEMP1 family is hugely diverse. As proteins that are constantly exposed to the immune system, selection pressure has caused them to expand into a large and antigenically variant protein family that varies significantly between different isolates [13,14]. The population of parasites released from the liver expresses most PfEMP1s, with a reduced repertoire found later in infection [22,23]. This maximizes the likelihood of establishing an infection regardless of the immune status or receptor availability of the host. Switching of PfEMP1-expressing genes then allows the parasite to adapt to immune challenge, increasing the likelihood of survival and passage to a new vector, perhaps most significantly during long periods of low transmission.

So to what degree is the PfEMP1 family too complex and too diverse to target therapeutically? Will it ever be possible to generate vaccine immunogens that induce a broadly inhibitory response against such a protein family? To answer these questions, we need to understand which PfEMP1s to target, due to their specific association with severe forms of malaria. We also need to understand their structures and to determine whether they retain the capacity to bind to specific endothelial receptors through conserved binding sites, and whether these sites can be mimicked by vaccine immunogens. If expression of specific PfEMP1s leads to the development of severe malaria, and if these PfEMP1s use conserved surfaces for ligand binding, then it may be possible to include their derivatives in future vaccines as components designed to reduce the incidence of death and severe disease.

## Which PfEMP1 proteins are linked to specific disease phenotypes?

Only a fraction of malaria cases develop severe symptoms. With many varied PfEMP1s in the genome, a major question has therefore been whether specific PfEMP1s, with specific receptor-binding phenotypes, are associated with particular severe disease syndromes. Indeed, PfEMP1 domains have been suggested to interact with a wide range of different human protein and carbohydrate ligands [24,25]. This raises several questions. Which are real, physiologically relevant interactions and which binding phenotypes are associated with particular severe malaria phenotypes? If such links can be demonstrated, then the component list for a PfEMP1-based vaccine would decrease.

The first PfEMP1 ligand-binding phenotype associated with a particular disease syndrome was found for pregnancy-associated malaria. Here, a single PfEMP1 in each parasite genome, known as VAR2CSA, was specifically expressed in parasites isolated from pregnant women suffering from malaria [26,27]. VAR2CSA mediates the sequestration of infected erythrocytes by binding to the chondroitin sulphates that fill the spaces of the placenta in contact with maternal blood [26,28]. With just one PfEMP1 associated with this form of the disease, and with immunity to pregnancy-associated malaria in multigravid women being associated with antibodies that bind VAR2CSA [29], this is a promising vaccine candidate and is being tested in clinical trials [30].

The association of specific PfEMP1s with severe malaria and the identification of their binding partners proved more challenging. Narrowing down the search required extensive sequence analysis and PfEMP1 domain classification [14]. First, the PfEMP1s were arranged into groups A, B and C, depending on their chromosomal location and the domain types encoded [31]. Expression of a subset of PfEMP1s, which contain a specific set of domains, known as CIDRα1 domains, was associated with severe malaria or brain endothelial cell binding [32–35]. Later, CIDRα1 domains were found to bind to human EPCR [16], thereby preventing EPCR from interacting with its natural ligand, activated protein C [16,36] (Figure 1). The blockage of EPCR-mediated signalling, through PfEMP1 binding, is proposed to lead to localized inflammation and to symptoms of severe disease [12,16,36,37].

While expression of EPCR-binding PfEMP1s has been associated with all forms of severe malaria [16,38–41], probably due to broad tissue tropism, efficient sequestration and the proposed pathogenic consequences of receptor binding, it is not specially linked to the development of cerebral disease. A particular association with cerebral symptoms was found for parasites expressing PfEMP1s that combine both an EPCR-binding CIDRα1 domain and a DBLβ domain that can bind to ICAM-1, allowing simultaneous binding of infected erythrocytes to both ligands [38,42,43] (Figure 1).

The links between these PfEMP1 types and severe or cerebral malaria are not absolute [16,38,44]. Nevertheless, expression of these PfEMP1s is a significant risk factor for the development of severe malaria symptoms and inhibitory antibodies targeting their respective receptor-binding domains are found in surviving patients [36,38]. While there is still much to discover about the PfEMP1s, these groups of domains are therefore highly promising candidates to target in attempts to reduce the devastating symptoms of severe malaria.

## Identifying conserved receptor-binding surfaces on PfEMP1s

The identification of groups of PfEMP1s [45] associated with specific disease syndromes is a significant step forward. But are these molecules suited for inclusion in a vaccine? The best example for progress towards an effective PfEMP1-based vaccine is VAR2CSA. Here, the immunogen was narrowed down to a ∼60 kDa fragment that has similar affinity and specificity for chondroitin sulphates as the full protein. Antibodies raised against this fragment recognize infected erythrocytes and block binding to chondroitin sulphate [46]. VAR2CSA is progressing through human clinical trials as a vaccine candidate [30].

However, VAR2CSA is not a classical PfEMP1 as it lies outside the normal organization of the protein family and is encoded by a single, relatively conserved gene in each parasite genome [26,27,45]. What about the more classical PfEMP1 family members? Do the ICAM-1 or EPCR-binding PfEMP1s, associated with severe disease, have the properties of vaccine immunogens? A combination of structural studies and extensive genomic analysis have revealed the degree to which these PfEMP1s remain conserved to allow receptor binding and has further identified regions of the ligand-binding domains that could be mimicked in immunogens [36,38,47] (Figure 1). The residues important for ICAM-1 binding in the DBLβ domains associated with cerebral malaria are remarkably conserved [38]. In contrast, EPCR-binding CIDRα1 domains are more complex, with little direct sequence conservation [36]. Despite this sequence diversity, however, the EPCR-binding surface is predominantly formed from a kinked helix that is conserved in shape and chemistry, indicating that it will be possible to design an immunogen that mimics these features and can induce cross-reactive antibodies. It is also expected that these features will remain conserved, to allow the CIDRα1 domains to continue binding to EPCR, limiting immune escape.

## From structural conservation to vaccine immunogen?

While there is still more to discover about the PfEMP1 family, recent studies have cut through some of their complexity, identifying binding phenotypes associated with severe disease syndromes and characterizing conserved structural features that mediate receptor binding. This revealed important principles that are likely to be shared by other PfEMP1s or other parasite surface protein families such as the RIFINs, which are less well studied, but have been suggested to contribute to the pathogenesis of malaria [48]. For example, genuine binding phenotypes are likely to be found in multiple family members and to be predicable from sequence when understood at a molecular level. In addition, conservation of shape and structure is likely to allow the maintenance of binding to invariant human receptors despite extensive sequence diversity.

A key question remaining is whether these insights can be used to design improved vaccine immunogens. Will knowledge of the chemically conserved surfaces that mediate receptor binding allow us to design molecules that mimic these regions? Will these molecules raise antibodies that specifically target the conserved chemistry of the receptor-binding sites and will these antibodies have broadly neutralizing potential?

Recent advances in structure-guided vaccine design raise hope that the key receptor-binding sites of PfEMP1 domains can be mimicked in designed immunogens. This could be achieved by grafting critical epitopes from PfEMP1 domains onto much smaller, weakly immunogenic scaffolds, a method that has been used successfully in viral vaccine development [49,50]. In the case of respiratory syncytial virus, the design of such immunogens, which specifically present epitopes for protective inhibitory antibodies, led to their re-elicitation [50]. The hope is that the use of similar design tools for a diverse protein family, like the PfEMP1s, will generate antibodies that specifically target functionally important and structurally conserved surfaces, blocking processes important for pathogenesis and reducing disease.

Many believe that surface protein families, such as the PfEMP1s, are simply too challenging to target by vaccines, with their depth of diversity so extensive that no immunogen will be able to raise antibodies that neutralize function across the whole family. Indeed, there is no doubt that it will be easier to generate broadly neutralizing antibodies that target more conserved proteins, such as the blood stage malaria antigen RH5 or the liver stage antigen CSP. However, for specific PfEMP1s, associated with particular binding phenotypes, there is hope. To retain the capacity to bind to their conserved human ligands, these PfEMP1s must retain the shape and chemical properties of their receptor-binding surfaces. The identification of interactions associated with severe disease, and the characterization of critical conserved features therefore provides the foundation required for these immunogen design studies. It will be fascinating to see the degree to which the latest tools in protein design can now allow the production of such immunogens and the degree to which they generate broadly reactive immune responses. If successful, such immunogens may form a valuable part of the multi-stage malaria vaccines of the future.

## Summary

- The malaria vaccines of the future are likely to be multi-component.
- Developing a component to prevent severe disease involves targeting parasite surface protein families such as PfEMP1.
- These are diverse and complex. However, recent studies have identified PfEMP1 associated with severe disease and have identified conserved ligand-binding surfaces.

## Abbreviations

CIDR: cysteine rich interdomain region
CSP: circumsporozoite protein
DBL: Duffy binding like
EPCR: endothelial protein C receptor
ICAM-1: intracellular adhesion molecule 1
PfEMP1: *P. falciparum* erythrocyte membrane protein 1.
